# Disturbed laterality of non-rapid eye movement sleep oscillations in post-stroke human sleep: a pilot study

**DOI:** 10.3389/fneur.2023.1243575

**Published:** 2023-11-30

**Authors:** Benjamin K. Simpson, Rohit Rangwani, Aamir Abbasi, Jeffrey M. Chung, Chrystal M. Reed, Tanuj Gulati

**Affiliations:** ^1^Department of Neurology, Cedars-Sinai Medical Center, Los Angeles, CA, United States; ^2^Department of Biomedical Sciences, Center for Neural Science and Medicine, Cedars-Sinai Medical Center, Los Angeles, CA, United States; ^3^Bioengineering Graduate Program, Department of Bioengineering, Henry Samueli School of Engineering, University of California, Los Angeles, Los Angeles, CA, United States; ^4^Department of Medicine, David Geffen School of Medicine, University of California, Los Angeles, Los Angeles, CA, United States

**Keywords:** stroke, sleep, EEG, spindles, slow wave oscillations, non-rapid eye movement sleep, delta waves

## Abstract

Sleep is known to promote recovery post-stroke. However, there is a paucity of data profiling sleep oscillations in the post-stroke human brain. Recent rodent work showed that resurgence of physiologic spindles coupled to sleep slow oscillations (SOs) and concomitant decrease in pathological delta (*δ*) waves is associated with sustained motor performance gains during stroke recovery. The goal of this study was to evaluate bilaterality of non-rapid eye movement (NREM) sleep-oscillations (namely SOs, *δ*-waves, spindles, and their nesting) in post-stroke patients vs. healthy control subjects. We analyzed NREM-marked electroencephalography (EEG) data in hospitalized stroke-patients (*n* = 5) and healthy subjects (*n* = 3). We used a laterality index to evaluate symmetry of NREM oscillations across hemispheres. We found that stroke subjects had pronounced asymmetry in the oscillations, with a predominance of SOs, *δ*-waves, spindles, and nested spindles in affected hemisphere, when compared to the healthy subjects. Recent preclinical work classified SO-nested spindles as restorative post-stroke and *δ*-wave-nested spindles as pathological. We found that the ratio of SO-nested spindles laterality index to *δ*-wave-nested spindles laterality index was lower in stroke subjects. Using linear mixed models (which included random effects of concurrent pharmacologic drugs), we found large and medium effect size for *δ*-wave nested spindle and SO-nested spindle, respectively. Our results in this pilot study indicate that considering laterality index of NREM oscillations might be a useful metric for assessing recovery post-stroke and that factoring in pharmacologic drugs may be important when targeting sleep modulation for neurorehabilitation post-stroke.

## Introduction

Stroke is a leading cause of motor disability world-wide. Despite advances in neurorehabilitation, there is a lack of widely adopted therapies that target plasticity post-stroke, and functional outcomes remain inconsistent (1–3). Sleep is known to play a major role in regulating plasticity (4–12) and accordingly, there has been an interest in modulating sleep for stroke motor rehabilitation (13, 14). To optimize efforts for effective sleep modulation, there is a need to better understand neural processing during sleep. Additionally, it is important to consider co-morbidities and concurrent pharmaceuticals that may impact excitatory/inhibitory neural transmission. Previous animal and human studies have shown that sleep can influence motor recovery post-stroke (2, 14–23), however more work is needed to understand how sleep neurophysiology is affected in stroke. This has become all the more important with advances in our understanding of sleep neurophysiology linking nested non-rapid eye movement (NREM) oscillations to plasticity, motor memory consolidation, and motor recovery (4, 6, 14, 24).

Sleep-dependent neural processing is crucial for memory consolidation, which is the process of transferring newly learned information to stable long-term memory (9, 25). Initial investigations looked at sleep’s role in declarative memory (26, 27), but recent studies have underscored sleep’s role in motor skill consolidation (5, 6, 28). Specifically, NREM sleep has been linked to the reactivation of awake motor-practice activity and performance gains in a motor skill after sleep (4–6). There is now a consensus that this consolidation occurs during temporal coupling of sleep spindles (10–16 Hz) to larger amplitude slow oscillations (SOs, 0.1–1 Hz) (6, 25, 29–31). Recent work in rodents has shown that these SOs nested with spindles decline immediately post-stroke and increase during motor recovery (14). This work also showed that delta waves (*δ* waves, 1–4 Hz), along with *δ* wave-nested spindles increased post-stroke and reduced during recovery. These two nested oscillations (namely, SO-nested spindles vs. *δ* wave-nested spindles) were shown to have a competing role during recovery. Pharmacological reduction of tonic γ-aminobutyric acid (GABA) neurotransmission shifted the balance toward restorative SO-nested spindles in the brain and increased the pace of recovery. The chief goal of our study was to see if NREM oscillations and their nesting were affected post-stroke in human patients within a hospital setting. Specifically, we wanted to check for laterality of NREM oscillations’ densities in stroke vs. contralateral hemisphere and compare it to healthy subjects.

Our study showed that, acutely post-stroke, there is an increase in SOs, *δ* waves, and spindles on stroke electrodes when compared to contralateral hemisphere electrodes, whereas healthy subjects had symmetrical density of these oscillations. Our linear mixed effect model revealed that there were significant fixed effects of stroke vs. contralateral electrodes for SOs and *δ* waves with overall medium effect sizes, including random effects of concurrent pharmacologic drugs. We also observed a large effect size of the linear mixed model for *δ* wave-nested spindles. Finally, we found that the proportion of SO-nested spindles to *δ*-wave-nested spindles was lower in stroke subjects compared to healthy subjects. Our work here in a pilot dataset suggests that laterality of NREM sleep oscillations could be a useful marker for physiological sleep activity post-stroke. Future work that confirms our findings in a larger dataset can inform acute stroke care management that also incorporates pharmacologic drug interactions and their effects on laterality of ‘restorative’ sleep oscillations.

## Patients and methods

This research was conducted in accordance with and approval of the Cedars-Sinai Medical Center Institutional Review Board (IRB). All research participants and/or their surrogates provided informed consent to participate in the study.

### Inclusion/exclusion criteria

Retrospective chart review of the Cedars Sinai EEG database was done to identify patients with acute middle cerebral artery strokes (MCA strokes; with high probability of stroke lesion affecting sensorimotor regions in the brain) who also received EEG monitoring as part of their hospital stay. We selected patients who received EEG in the acute period (2–3 days) post-stroke. Other inclusion criteria were that this should be the first stroke for the patient, they should be within 50–80 years of age, and the patients should not have any sleep disorders or circadian /diurnal rhythm disruption. Subjects were excluded if they were pregnant or diagnosed with uncontrolled medical conditions. Five patients were retrospectively identified for this study, with notable limited availability of EEG studies done within 2–3 days after an MCA distribution stroke. Of the 5 patients, 3 were female and 2 males, all within the age range of 50–80 years old (see Table 1 for other details regarding demographic and clinical information). Indications for EEG were universal for altered mental status after acute stroke. P1 was noted to be on continuous infusion of propofol (<10 mcg total) and infusions of dexamethasone every 4 h. P2 and P5 were treated with levetiracetam 500 mg twice daily. P2 was also on acyclovir which was discontinued after cerebrospinal fluid (CSF) evaluated negative for meningitis; and P5 was administered nonepinephrine due to being in shock acutely and improved within 24 h. P3 and P4 were not given propofol, dexamethasone, or levetiracetam. Unlike all other patients, P4 had subcortical involvement in stroke. It is important to note that spindle oscillations are postulated to have a subcortical (thalamocortical) origin (32). P5 had a hemorrhagic stroke (ruptured right MCA aneurysmal stroke). P2 had partial status epilepticus involving the right temporal lobe. We excluded seizure related epochs based on manual inspection of recordings. This inspection was done by epileptologist (C.M.R.) and seizures were excluded based on no evolving seizure pattern across electrodes (10–20 EEG system). Hence, all our presented data was from sleep periods in all the five patients (even in the patient with status epilepticus). An average of ~5.98 ± 1.26 h [or 358.80 ± 75.40 min, mean ± standard error of mean (s.e.m.)] of NREM sleep was identified and analyzed in each of the five patients. We were not able to analyze REM/ wake periods in these recordings due to the lack of EMGs/ video recordings. Additionally, healthy subjects’ dataset from Cox *et al*., *Sleep Medicine Reviews*, 2020 (33, 34) with average NREM sleep of 3.07 ± 0.14 h (or 183.91 ± 8.38 min) was analyzed for 3 subjects.

**Table 1:** Patient clinical information.

Patient	P1	P2	P3	P4	P5
Age	56	68	51	56	52
Sex	F	F	M	M	M
Race/ethnicity	Hispanic	White/Caucasian	Hispanic	Black/African-American	White/Caucasian
Stroke location	R MCA	R MCA	L MCA	R MCA	R MCA
NIHSS	3	N/A	21	N/A	N/A
Time of recording after stroke	2 days	2 days	3 days	3 days	3 days
Comorbidities	COVID	Partial status epilepticus (right temporal)	ESRD, HFrEF	Pituitary macroadenoma, Central hypoT	Ruptured R MCA aneurysm
Sleep disorders (e.g., obstructive sleep apnoea)	No	No	No	No	No
Circadian rhythm disruption	No	No	No	No	No
Alcohol	Yes	No	N/A	No	No
Smoking	No	No	N/A	No	No
Rx (concurrent)	Propofol gtt, Dexamethasone, Remdesivir	Levetiracetam, Acyclovir, Vancomycin, Cefepime	ASA/Plavix	ASA, Levothyroxine	Levetiracetam, Levophed

From top to bottom, information for five patients P1–P5. Patient age, sex, race/ethnicity, stroke location, NIHSS, days from stroke when the EEG data was acquired, associated co-morbidities, sleep disorders, circadian rhythm disruption, alcohol and smoking substance consumption status, and concurrent medications during EEG recording are specified. NIHSS, National Institutes of Health Stroke Scale; R/L MCA, Right/left middle cerebral artery; COVID, Coronavirus disease - 2019; ESRD, End-stage renal disease; HFrEF, Heart failure with reduced ejection fraction; HypoT, hypothyroidism; ASA, Acetylsalicylic Acid (Aspirin); N/A, not available. Patient groups: **blue**: patients in propofol medication group (Group-1); **orange**: patients in levetiracetam medication group (Group-2); **green**: patients in other medication group (Group-3).

### EEG analysis and identification of NREM oscillations

Patients with overnight EEG recordings 2 to 3 days post-stroke were included. The data, obtained by a Natus Xltek EEG and Sleep System, was de-identified and made compatible for analysis with MATLAB. Each 30-s epoch was manually marked for NREM sleep by an expert scorer (C.M.R. and B.K.S.). EEG epochs were analyzed for NREM sleep in a bipolar montage. In the stroke patients, the following analyses were done with EEG data in a referential montage, referenced to the auricle electrodes. Spindles, SOs, and *δ* waves were extracted from these NREM epochs using custom code in MATLAB (details below). This allowed for the identification of specific sleep waveforms and how they nested temporally and topographically during NREM sleep. We assessed spindles and their nesting to SOs and *δ* waves. Topographical maps of the average density of these sleep oscillations allowed us to visualize the average densities with respect to electrode location, especially their lateral symmetry between hemispheres.

From the healthy subjects dataset, we used the common linked mastoids referenced data (33) and analyzed NREM sleep. We selected 20 electrode channels in similar locations as stroke patient data for further analysis (because the healthy subject data had more electrodes than stroke patient dataset). Similar to stroke EEG data; spindles, SOs, and *δ* waves were extracted from these NREM epochs using custom code in MATLAB and analyzed.

#### EEG data processing

**Figure 1:**
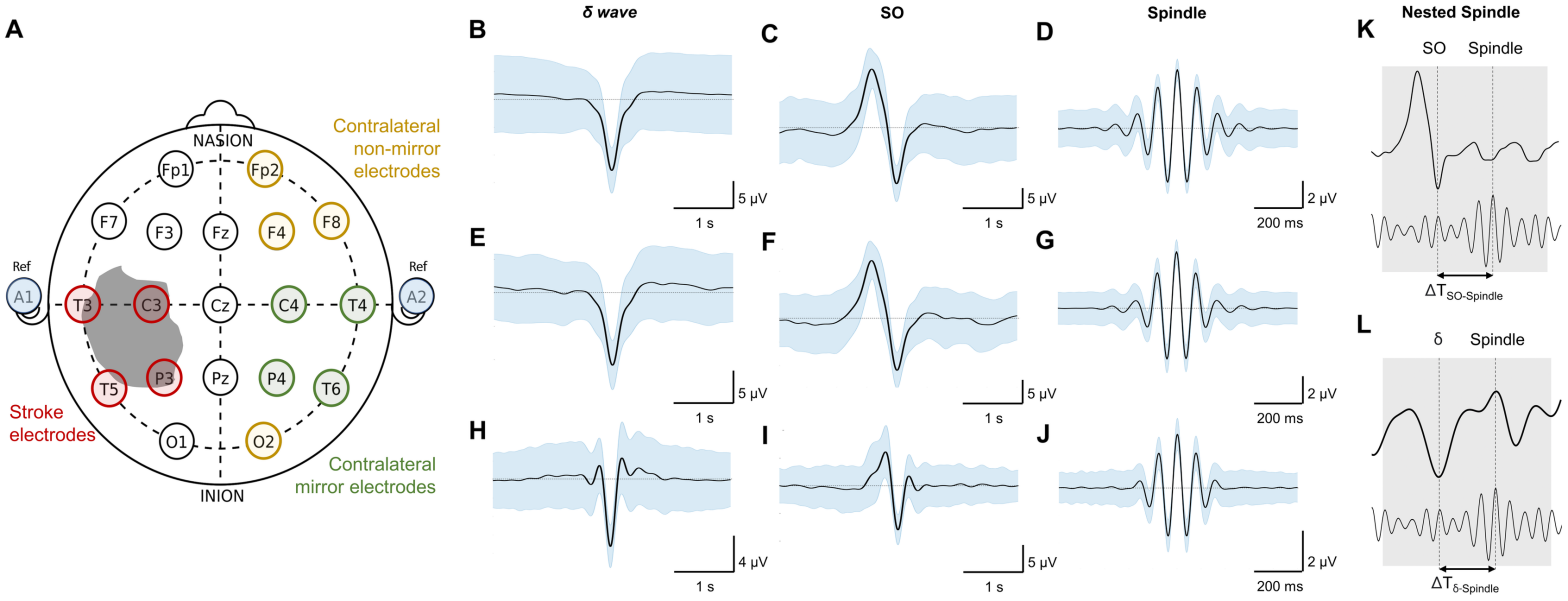
Stroke vs. contralateral mirror/non-mirror electrode assignment and NREM sleep oscillations. **(A)** 10–20 system for EEG (used in stroke patients) showing locations of all electrode locations recorded with an illustration of stroke. Gray shaded area shows a representative stroke perilesional region. Blue shaded circles represent auricular electrodes (A1, A2) that were used for referencing in the stroke patients. Red circles indicate identified *stroke electrodes* based on proximity to the perilesional area. Green circles indicate identified *contralateral mirror* (CM) *electrodes* which are contralateral and mirrored to identified *stroke electrodes*. *Yellow* circles indicate identified *contralateral non-mirror* (CNM) *electrodes* which are electrodes other than *contralateral mirror (CM) electrodes* in non-stroke hemisphere. **(B)** Mean *δ–*wave along with s.e.m. (standard error of mean) bands (blue) for all identified *δ–*waves from an example *stroke electrode* channel from EEG data recording for one stroke patient. **(C)** Same as **(B)** for SO waveforms. **(D)** Same as **(B)** for spindle waveforms. **(E–G)** Same as **(B–D)** for one example *contralateral mirror* electrode channel for a stroke patient. **(H–J)** Same as **(B–D)** for one example channel for a healthy subject. All waveforms are centered around the detected states. **(K)** Illustration of SO-spindle nesting. Nesting window was −0.5 to +1.0 s from SO’s UP state as shown. **(L)** Illustration of *δ–*wave-spindle nesting. Nesting window was −0.5 to +1.0 s from *δ* UP state as depicted.

For stroke patients, NREM-marked EEG data from all channels was referenced with respect to the average of the auricular electrodes (A1 & A2, Figure 1A) while the heathy control dataset had common linked mastoids referenced EEG data. Any high amplitude artifact in the differential EEG signal was removed. We utilized previously-used methods for automatic detection of these NREM oscillations (6, 14, 35). For *δ/SOs detection*, signal was first passed through a 0.1 Hz high-pass filter and then a 4 Hz low-pass Butterworth filter. All positive-to-negative zero crossings, previous peaks, following troughs, and negative-to-positive zero crossings were identified. A wave was considered a *δ* wave if its trough was lower than the negative threshold and preceded by a peak that was lower than the positive threshold, within 500 ms (Figures 1B,E,H). SOs were classified as waves with troughs lower than a negative threshold (the bottom 40 percentile of the troughs) and preceding peaks higher than a positive threshold (the top 15 percentile of the peaks; Figures 1C,F,I). Duration between peaks and troughs was between 150 ms and 500 ms. For *spindle detection*, EEG data was filtered using a 10 Hz high-pass Butterworth filter and a 16 Hz low-pass Butterworth filter. A smoothed envelope of this signal was calculated using the magnitude of the Hilbert transforms with convolving by a Gaussian window (200 ms). Epochs with signal amplitude higher than the upper threshold (mean, μ + 2.5* standard deviation (s.d.), σ) for at least one sample and amplitude higher than the lower threshold (μ + 1.5*σ) for at least 500 ms were considered spindles (Figures 1D,G,J). The lower threshold was used to define the duration of the spindle. Nested SO-spindles (parallel to *k*-complexes studied in humans) were identified as spindle peaks following SO peaks within 1.5 s duration (Figure 1K). The same criterion was used to identify *δ* wave-nested spindles (Figure 1L).

#### Data analysis

**Figure 2:**
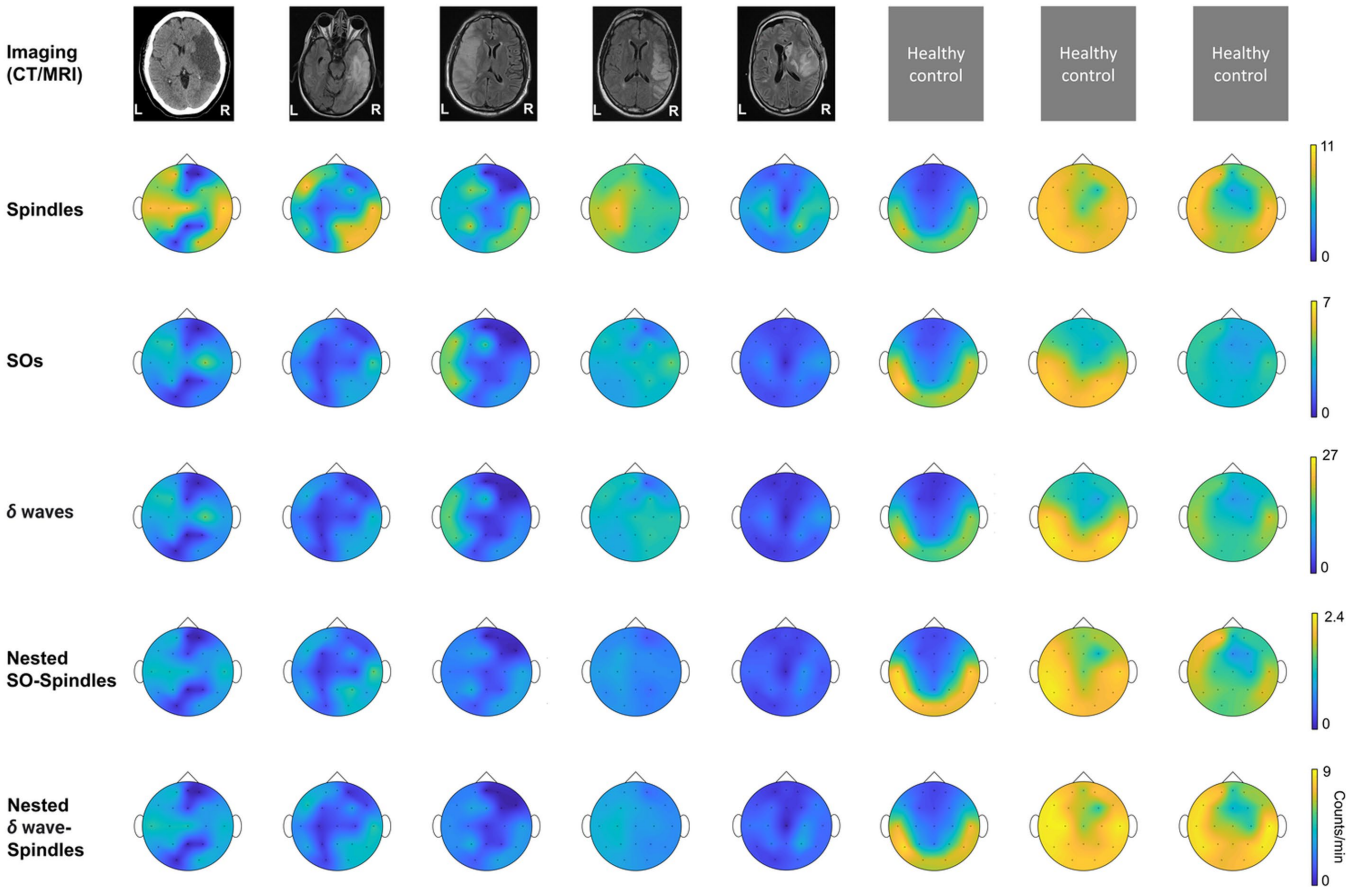
Imaging data and topographical density plots for different NREM oscillations. Top to bottom: *Imaging data*: CT (computed tomography) image for patient P1, T2 sequences of MRI (magnetic resonance imaging) images for patients P2–P5; no imaging data available for healthy subjects (P6–P8). Radiologic imaging has been flipped horizontally to align with topographic density maps; i.e., image left, and right are ipsilateral to patient left and right. Left and right are marked in imaging figures (P1–P5) and apply to density topographical maps below them; *Topographical maps* for detected *spindle* density (count/min) during NREM sleep for all subjects; *Topographical maps* for detected *SO* density (count/min) during NREM sleep for all subjects; *Topographical maps* for detected *δ waves’* density (count/min) during NREM sleep for all subjects; *Topographical maps* for detected *nested SO-spindle* density (count/min) during NREM sleep for all subjects; *Topographical maps* for detected *δ wave-nested*-*spindle* density (count/min) during NREM sleep for all subjects. Color map shown at right for all the panels in a row.

We generated topographical maps of these different waveforms using *plot_topography* function in MATLAB (36) as shown in Figure 2. The patients were separated into three groups based on concurrent medications, as detailed in Table 1. Patient 1, assigned to Group 1, was on continuous propofol and dexamethasone injections every 4 h. Group 2 (patients 2 and 5) was administered levetiracetam (Keppra) twice daily; and Group 3 (patients 3 and 4) was not on medications known to significantly modulate excitatory/inhibitory neural transmission.

Perilesional electrodes were identified by analyzing post-stroke magnetic resonance imaging (MRI) and computer tomography (CT) neuroimaging. We marked *Stroke electrodes* as the electrodes covering the perilesional region of the brain as shown in Figure 1A. The mirror opposite electrodes on the contralateral side were marked as *Contralateral mirror (CM) electrodes* for further analysis (Figure 1A). The non-mirror opposite electrodes on the contralateral side were marked as *Contralateral non-mirror (CNM) electrodes*.

**Figure 3:**
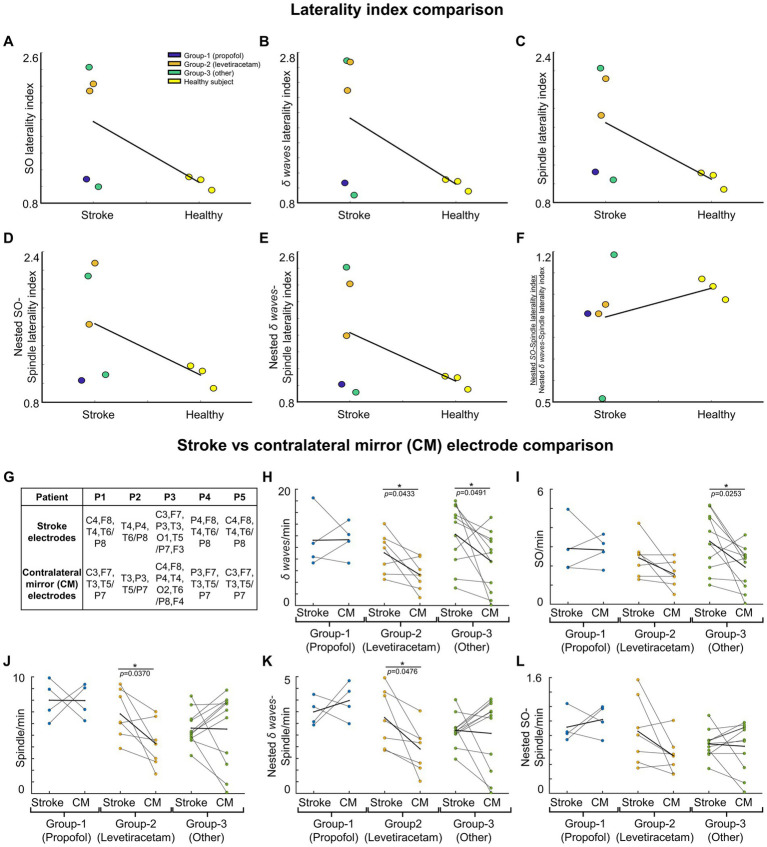
NREM oscillations’ laterality in stroke patient’s vs. healthy controls; and NREM oscillations’ densities for different patient groups on stroke verses contralateral mirror (CM) electrodes. For stroke patients’ laterality index (LI) is defined as ratio of mean of stroke electrode NREM densities to all contralateral electrode NREM densities. For healthy subjects’ laterality index is defined as ratio of mean of left hemisphere electrode NREM densities to right hemisphere electrode NREM densities. **(A)** LI for SO density for stroke patients and healthy controls. Black line connects the mean of stroke and control group. Dots represent different patients/subjects; blue dots: Patients in propofol medication group; orange dots: Patients in levetiracetam medication group; green dots: Stroke patients in other medication group; yellow dots: Healthy subjects. **(B)** Same as **(A)** for *δ* wave density LI. **(C)** Same as **(A)** for spindle density LI. **(D)** Same as **(A)** for nested SO-spindle density LI. **(E)** Same as **(A)** for Nested *δ* wave-spindle density LI. **(F)** Ratio of LI for nested SO-spindle density and nested *δ* wave-spindle density. **(G)** Table showing selected *stroke* and *contralateral mirror electrodes* (CM) for all patients. **(H)** Comparison of *δ wave* density (count/min) on *stroke* vs. *CM electrode*s for patients on different medications. Thick black line shows the mean values within the group. Thinner black lines join pair of stroke and CM electrode. Dots represent the NREM oscillations’ density for single electrode. **(I)** Same as **(H)** for SO density. **(J)** Same as **(H)** for spindle density. **(K)** Same as **(H)** for nested *δ* wave-nested spindle density. **(L)** Same as **(H)** for SO-nested spindle density. *statistically significant *p* values for two-tailed *t*-test.

We compared the symmetry in NREM oscillations’ density across hemispheres for stroke patients and healthy control using a laterality index (Figures 3A–F). Laterality index of 1 meant the average density being analyzed for electrode locations selected across hemisphere is equal. For stroke patients, laterality index was defined as the ratio of mean of stroke electrodes’ NREM densities to all contralateral electrodes’ NREM densities. For healthy subjects, laterality index was defined as the ratio of the mean of left hemisphere electrodes’ NREM densities to right hemisphere electrodes’ NREM densities. We also compared the ratio of SO-nested spindles laterality index to *δ* wave-nested spindles laterality index for stroke vs. healthy subjects.

### Statistical analysis

We performed a linear mixed effect analysis for all patients comparing the *Stroke electrodes* density vs. Co*ntralateral (CM/CNM) electrodes* density for different waveforms using the *fitlmematrix* function in MATLAB. The linear mixed effect model was fitted by maximum likelihood using the formula below (1) for all the different waveforms identified during EEG data processing. Medication groups were defined as the three groups mentioned earlier. This model considered fixed effects of stroke vs. contralateral (CM/CNM) electrodes, and the random effect of electrodes and medication groups depending on the patient and was represented as:

*Waveform Density ~ Intercept + Electrode + (Intercept + Electrode + Medication Groups | Patient)*.

The above formula/equation is written in a format like the documentation for *fitlmematrix* Matlab function. We compared the *Stroke electrodes* density vs. *contralateral (CM/CNM) electrodes* density within each medication group using a two-tailed *t*-test. Contralateral electrodes chosen were mirrored electrodes (Figures 3G–L) or non-mirrored (Supplementary Figures S2A–F). One-way ANOVA was used to compare the stroke electrodes’ NREM oscillations’ density of the three different medication groups.

We calculated r-squared (*R*^2^) and the Cohen’s *d* values for the overall linear mixed effect model generated. However, the *p*-values were specifically assessed for fixed effect of electrodes (stroke vs. CM/CNM). Cohen’s *d* was used to evaluate if the nested data (all data combined) for NREM oscillations had a small, medium or large experimental effect (Cohen’s *d* = 0.20, 0.50 or 0.80, respectively) (37). Effect size indicates if research findings have practical significance. Metrics such as Cohen’s *d* are better at the planning stage for pilot studies, like the one here, to determine optimal sample sizes for sufficient power in bigger clinical trials (38). We summarized the linear mixed effects models results in the tables in the Supplementary Information (Supplementary Tables S1, S2).

## Results

One of the limitations of retrospectively analyzing EEG data gathered from clinical EEG was the heterogeneity encountered across the subjects studied, a contrast from the controlled setting of related rodent studies. With this in mind, we noted that one important similarity across the study population was the indication for EEG: concern for underlying seizure in the setting of altered mental status and recent hemispheric stroke. Accordingly, the patients were all hospitalized, and our analysis benefited from close pharmacologic documentation. We observed differences in laterality of NREM oscillations in stroke patients. We observed higher SOs, *δ* waves, spindles and spindles nested to SOs and *δ* waves in the stroke hemisphere. For the patient with subcortical involvement in stroke, we observed a decrease in spindles in the stroke hemisphere. We also observed effects of concurrent medications, particularly medications that might influence neural transmission.

### NREM oscillation densities symmetry is disturbed acutely in stroke

We found that stroke patients had laterality differences (higher or lower densities in stroke hemisphere) for all NREM oscillations, while the healthy subject NREM oscillation density looked more symmetrical across hemispheres (Figure 2). Comparing the laterality index (LI) (as defined in methods), we found that the LI was closer to 1 on average with low variance for healthy subjects. For stroke patients, LI was higher than 1 on average with high variance. SO density LI’s were: stroke: 1.78 ± 0.34 and healthy: 1.05 ± 0.06 (Figure 3A). *δ wave* density LI’s were: stroke: 1.93 ± 0.44 and healthy: 1.05 ± 0.06 (Figure 3B). Spindle density LI’s were: stroke: 1.65 ± 0.27 and healthy: 1.05 ± 0. 0.07 (Figure 3C). SO-nested spindles LI’s were: stroke: 1.63 ± 0.30 and healthy: 1.09 ± 0.09 (Figure 3D). *δ* wave-nested spindles LI’s were: stroke: 1.63 ± 0.34 and healthy: 1.05 ± 0.06 (Figure 3E). The ratios of nested SO-spindles LI’s and *δ* wave-nested spindle LI’s were: stroke: 0.90 ± 0.12 and healthy: 1.03 ± 0.03 (Figure 3F).

### SO and *δ* wave density increased in perilesional electrodes

Next, we wanted to look at stroke-affected electrodes in stroke patients vis-à-vis the contralateral hemisphere electrodes. In the contralateral hemisphere, we looked at mirrored electrodes (CM, as defined in the methods; Figure 3G), or non-mirrored electrodes (CNM, as defined in methods; Supplementary Figure S2A). Consistent with previous reports, we found that stroke electrodes had increased low-frequency (< 4 Hz) oscillations (Figures 3H,I; Supplementary Figures S2B,C) (39). Our mixed-effects model showed a significant fixed effect of stroke vs. CM and CNM electrodes for a subset of NREM oscillations and overall medium to large effect sizes which included random effects of concurrent pharmaceuticals. We observed higher *δ* wave density in the perilesional electrodes (Figure 3H; Supplementary Figure S2B; Supplementary Tables S1, S2 provide statistical details for stroke vs. CM or CNM: *p*-value is provided for the fixed effect (“electrode”), *R*^2^ and Cohen’s *d* are for the overall model with fixed and random effects, conventions same henceforth). Our comparison of LI’s of SOs and *δ* wave showed that LI’s were higher in stroke patients compared to healthy subjects: Mean LI’s for SOs were: stroke: 1.78 ± 0.34 and healthy: 1.05 ± 0.06; mean LI’s for *δ* wave were: stroke: 1.91 ± 0.44 and healthy: 1.05 ± 0.06. We also observed that Group-1 (propofol and dexamethasone) and Group-3 (others) both had higher *δ* wave density on stroke electrodes than Group-2 (levetiracetam) (Figure 3H; Supplementary Figure S2B; stroke electrodes’ *δ* wave density- Group 1: 11.23 ± 2.53 counts min^−1^ (mean ± s.e.m.); Group 2: 9.07 ± 1.32 counts min^−1^; Group 3: 12.25 ± 1.59 counts min^−1^, see Supplementary Table S3 for details). Group-2 and Group-3 showed a high density of *δ* waves in the stroke electrodes vs. CM/CNM electrodes (Figure 3H; Supplementary Figure S2B). For SOs, there was a significant fixed effect of stroke vs. contralateral electrodes (Figure 3I; Supplementary Figure S2C; Supplementary Tables S1, S2 provide *p*-value and Cohen’s *d*). We observed that the patients in Group-1 did not show a significant difference between stroke or contralateral electrode SO density, while patients in Group-2 showed elevation in SO on stroke electrodes when compared to CM electrodes (Figure 3I). The patients in Group-3 showed increased SOs on stroke electrodes when compared to CM/CNM electrodes (Figure 3I; Supplementary Figure S2C; stroke electrodes’ SO density: Group 1: 2.91 ± 0.71 counts min^−1^; Group 2: 2.42 ± 0.37 counts min^−1^; Group 3: 3.29 ± 0.45 counts min^−1^; see Supplementary Table S3 for details).

For spindle oscillations, LI’s were higher in stroke patients (Mean LI spindles, stroke: 1.65 ± 0.27 and healthy: 1.05 ± 0. 0.07). Interestingly, in one patient with subcortical involvement with stroke (P4), spindles were higher in the contralesional hemisphere (Figure 3J). Linear mixed-effects model did not show a significant fixed effect for spindle density on stroke vs. contralateral electrodes; overall, it was a medium effect size based on the Cohen’s *d* (Figure 3J; Supplementary Figure S2D; see Supplementary Tables S1, S2 for *p*-value and Cohen’s *d*). Spindle density was found to be the highest on the stroke electrodes in the patient in Group-1 (8 ± 0.88 counts min^−1^), followed by the patients in Group-2 (6.83 ± 0.79 counts min^−1^), and then patients in Group-3 (5.61 ± 0.44 counts min^−1^) (Figure 3J; Supplementary Figure S2D; see Supplementary Table S3 for details).

### *δ* wave-nested spindles and SO-nested spindles

Next, we looked at nested oscillations, namely *δ* wave-nested spindles and SO-nested spindles oscillations that were recently shown to have a competing role in memory consolidation and inverse trend during stroke recovery (6, 14). LI’s for both nested oscillations were observed to be higher in stroke subjects. Mean LI’s for SO-nested spindle were: stroke: 1.64 ± 0.29 and healthy: 1.09 ± 0.09; and mean LI’s for *δ* wave-nested spindle were: stroke: 1.63 ± 0.34 and healthy: 1.05 ± 0.06. Linear mixed effects models of *δ* wave-nested spindles and SO-nested spindles did not show a significant difference between stroke and contralateral electrodes, whereas these models still had large and medium effect sizes, respectively (Supplementary Tables S1, S2; Figure 3K; Supplementary Figure S2E, *δ* wave-nested spindle density on stroke electrodes: Group-1: 3.49 ± 0.30 counts min^−1^; Group-2: 3.25 ± 0.48 counts min^−1^; Group-3: 2.70 ± 0.20 counts min^−1^, also see Supplementary Table S3; *SO*-nested spindle density on stroke electrodes: Group 1: 0.92 ± 0.11 counts min^−1^; Group 2: 0.86 ± 0.17 counts min^−1^; Group 3: 0.68 ± 0.06 counts min^−1^; see Figure 3L; Supplementary Figure S2F; Supplementary Table S3). Notably, the ratios of SO-nested spindle LI’s to *δ* wave-nested spindle LI’s were lower in stroke subjects compared to heathy subjects (Mean LI ratio, stroke: 0.0 ± 0.12 and healthy: 1.03 ± 0.03). This might indicate relatively increased *δ* wave-nested spindles when compared to SO-nested spindles (the oscillations that have a competing role in forgetting vs. strengthening, respectively) in the perilesional areas for stroke brain when compared to healthy brain.

Together, the results in this limited dataset showed that lateral symmetry of NREM oscillations is disturbed in stroke (Figures 3A–F), when compared to healthy subjects. These results also indicated that there is an elevation of SO, *δ* wave, spindles, and spindle nesting to SOs or *δ* waves in the perilesional areas post-stroke. Future work can confirm these findings on laterality of sleep oscillations in a larger dataset that also considers the pharmacologic drug interactions.

## Discussion

Our results show that, post-stroke there is a disturbance in laterality of NREM sleep oscillations across ipsilesional and contralesional hemispheres. Interestingly, hemispherical differences in these nested oscillations were less pronounced in healthy subjects, and oscillations appeared mostly symmetric. We used a laterality index for comparing NREM oscillations, with an emphasis on nested oscillations, i.e., *SO*-nested spindle oscillations and *δ* wave-nested spindle oscillations. Our results here can be a precursor to future investigations studying neuromodulation of sleep for rehabilitation. While our findings are preliminary in a small pilot dataset, they report an interesting effect size, suggesting a roadmap for delineating pathological sleep in larger cohorts and optimal therapeutic modulation to promote recovery.

### Sleep and plasticity post-stroke

Preclinical and clinical studies that have evaluated local-field potentials (LFPs) in animals (40, 41) and EEG in human patients (22, 42, 43) have found increased low-frequency power during awake, spontaneous periods after a stroke. These studies postulate that this increased low-frequency activity could be a marker of cortical injury and loss of subcortical inputs (44). Our findings on increased SOs and *δ* waves on stroke electrodes are indicative of similar phenomena. We also found an increase in SO-nested spindles and *δ* wave-nested spindles on stroke electrodes along with a lower ratio of SO-nested spindle LI’s to *δ* wave-nested spindle LI’s (Figure 3F). There is growing evidence that temporal coupling of spindles to SOs is a primary driver of sleep-related plasticity and memory consolidation (6, 30, 31, 45–48). SO-nested spindles are linked to spike-time dependent plasticity (49). These events are also related to reactivation of awake experiences (30, 47, 50). Importantly, disruption of this coupling can impair sleep-related memory consolidation of awake experiences (6). This same work showed that SO-nested spindles and *δ* wave-nested spindles compete to either strengthen or forget a memory. Our results indicate that balance of SO-nested spindle density and *δ* wave-nested spindle density is disturbed across hemispheres in stroke patients compared to healthy subjects. These disruptions might be related to impaired sleep-processing that impact recovery. Interestingly, we observed large to medium effect sizes in our linear mixed-effects models for *δ* wave-nested spindle and SO-nested spindle where we considered fixed effects of electrodes and random effects of drugs and patients. It is worth noting that drugs like propofol can impact such nested sleep oscillations (51, 52). It may be important to consider the effects of drugs on sleep oscillations when modulating sleep for stroke recovery.

### Propofol and levetiracetam: effect on sleep

We made observations on different medications that stroke patients received during sleep EEG recordings. Group-1 received propofol, which is one of the most commonly used anesthetics in neurologic intensive care units after stroke or traumatic brain injury (53). It exerts its action by potentiating the activity of chloride currents through GABA receptors while blocking voltage-gated sodium channels (54–56). The patient on propofol received less than 10 mcg dose of propofol which is not known to impact sleep (57, 58). Group-2 received levetiracetam (Keppra), which is a newer anti-seizure drug. The exact mechanism for its anti-seizure function is unclear, but it is believed to exert its effect through synaptic vesicle glycoprotein 2A (59). Through this mechanism, levetiracetam is capable of modulating neurotransmission by inhibiting calcium currents (60). A study has shown that levetiracetam has minimal effects on sleep parameters like total sleep duration, sleep latency, and sleep efficiency in both healthy humans and partial epilepsy patients (61). However, observations have been made that levetiracetam can reduce motor activity and cause daytime drowsiness in patients (61, 62). Propofol, by its GABAergic action, causes greater loss of faster frequencies during induction with a shift in alpha frequencies to the frontal regions that reverses post-awakening (63–65). Since our linear mixed-effects model had large to medium effect sizes when considering random effects of drugs on all NREM oscillation, it may be useful to explore the impact of drugs on NREM sleep densities with larger patient cohorts in the future.

### Sleep processing and stroke rehabilitation

Recent rodent work profiled SO-nested and *δ* wave-nested spindles during the course of stroke recovery and found links between these nested structures and motor performance gains during recovery (6). This work specifically looked into reach task, but clinical rehabilitation approaches can be varied (66–68). It is likely that the sleep features of nested oscillations and their putative pathological or physiological roles need to be factored in when considering timing for rehabilitation, irrespective of training type. Previous human and rodent studies have also suggested critical periods in training that can offer long-term benefits (69–71). Past studies that have found low-frequency power in awake state in stroke patients might be related to our findings of increased SO and *δ* waves densities. Future studies where EEG data is captured over longer periods may delineate a transition of *δ* wave LI, SOs LI, *δ* wave-nested spindles LI (pathological sleep) and SO-nested spindle LI (physiological sleep), and its relation to critical periods post-stroke for optimal timing of rehabilitation. For example, SO-nested spindles LI and *δ* wave-nested spindles LI proportions between hemispheres could be targeted to be brought closer to unity as in healthy subjects, to accelerate recovery.

### Modulation of sleep as a therapeutic intervention

The results we have presented can form the basis of translational studies in the future that target modulation of sleep post-stroke. Animal studies have suggested that modulation of GABAergic transmission (specifically GABA_A_-receptor mediated tonic inhibition) in the perilesional cortex can serve as a therapeutic target to promote recovery, and that blocking of GABA_A_-mediated tonic inhibition promoted motor recover maximally in the first 1 to 2 weeks post-stroke (72, 73). Both short-term (acute) and long-term chronic infusion of GABA_A_ inhibiting compounds have been tested, and long-term infusion was shown to be better (72). Long-term pharmacologic modulation, as shown by Clarkson and colleagues, may be essential to achieve observable motor benefits in human patients. Benefits of long-term infusion include the effect of the drug not only with rehabilitation-specific online (awake) training, but also during offline memory consolidation during sleep.

Studies such as ours can also help guide electric stimulation-based neuromodulation for augmenting recovery. SOs and *δ* waves can be easily monitored using EEG in stroke patients. Non-invasive brain stimulation during sleep (30, 47, 74, 75) can be used to modulate specific NREM oscillations. Invasive stimulation approaches, such as epidural stimulation (76), can also focus on sleep state to optimize sleep neural processing. Similar approaches have shown that direct epidural motor cortical electric stimulation can enhance awake performance and neural activity (77, 78) and epidural stimulation of subcortical regions can also modulate low-frequency oscillations in the motor cortex (79). However, such approaches have not been applied during sleep. A recent study suggested that modulating UP states during sleep can enhance recovery (18). It is plausible that future approaches targeting sleep, when delivered in a closed-loop fashion, optimize both awake task performance and its consequent sleep processing, and may lead to greater long-term benefits during rehabilitation. Indices such as laterality index that we pursued here may serve a utilitarian purpose in long-term sleep evaluation post-stroke with different treatments. Our pilot observations here also suggest that concurrent pharmacologic drugs may affect NREM oscillations. Future work can confirm these effects in larger cohorts and if medication effects should be considered when personalizing sleep stimulation.

### Limitations

One of the limitations of our study is the lack of a link between sleep architecture and motor status. Future work that studies sleep over longer periods post-stroke and assesses motor functionality longitudinally may find more robust links between sleep processing and related gains in motor performance. It is also possible that, with more effective task performance and associated awake neural dynamics (77, 78, 80), efficacy of sleep may change. Precise disruption of sleep processing, specifically SO-spindle coupling in healthy animals, was sufficient to prevent offline performance gains, even when awake task learning was robust (6). This work also showed that precise modulation of the extent of sleep spindle-SO coupling in healthy animals could either enhance or impede sleep processing. While extension of this work in stroke animals has shown SO-spindle nesting resurges with recovery (14), future animal studies that modulate sleep microarchitecture can study if artificial manipulation of SO-nested spindles or *δ* wave-nested spindles after stroke are sufficient to enhance or impair motor recovery. Our work here showed that both SO-nested spindles and *δ* wave-nested spindles increased in stroke affected hemisphere acutely post-stroke. Future work that monitors these oscillations for longer periods can assess if SO-nested spindles should increase with respect to *δ* wave-nested spindles for better recovery in human stroke patients.

As a pilot retrospective study, one more limitation is a smaller sample size with varying lesion location and volume. While we focused on getting patients with cortical lesions and MCA involvement, sleep may have been impacted differently for one patient with a primarily subcortical stroke. For example, a stroke in the white matter that impacts thalamocortical networks may also impact spindles. Future work with larger sample sizes and incorporation of motor task rehabilitation training and drug manipulation, may provide stronger links to engineer sleep to benefit motor recovery post-stroke.

## Data availability statement

All requests for raw and analyzed data and materials are promptly reviewed by the Cedars-Sinai Medical Center to verify if the request is subject to any intellectual property or confidentiality obligations. Patient-related data may be subject to patient confidentiality. Requests to access the datasets should be directed to TG at tanuj.gulati@csmc.edu.

## Ethics statement

The studies involving humans were approved by Cedars-Sinai Institutional Review Board. The studies were conducted in accordance with the local legislation and institutional requirements. The participants provided their written informed consent to participate in this study.

## Author contributions

BS, RR, CR, and TG contributed to the design of the study, interpretation of the data, and draft of the article. RR, BS, and AA contributed to the analysis of the data. BS, JC, and CR contributed to the acquisition of data. All authors contributed to the article and approved the submitted version.
